# Molecular Regulation of Fetal Brain Development in Inbred and Congenic Mouse Strains Differing in Longevity

**DOI:** 10.3390/genes15050604

**Published:** 2024-05-09

**Authors:** Maliha Islam, Susanta K. Behura

**Affiliations:** 1 Division of Animal Sciences, University of Missouri, Columbia, MO 65211, USA; 2 MU Institute for Data Science and Informatics, University of Missouri, Columbia, MO 65211, USA; 3 Interdisciplinary Reproduction and Health Group, University of Missouri, Columbia, MO 65211, USA; 4 Interdisciplinary Neuroscience Program, University of Missouri, Columbia, MO 65211, USA

**Keywords:** gene expression, epigenetics, genetic variation, splice variation, brain development, longevity

## Abstract

The objective of this study was to investigate gene regulation of the developing fetal brain from congenic or inbred mice strains that differed in longevity. Gene expression and alternative splice variants were analyzed in a genome-wide manner in the fetal brain of C57BL/6J mice (long-lived) in comparison to B6.Cg-*Cav1*^tm1Mls^/J (congenic, short-lived) and AKR/J (inbred, short-lived) mice on day(d) 12, 15, and 17 of gestation. The analysis showed a contrasting gene expression pattern during fetal brain development in these mice. Genes related to brain development, aging, and the regulation of alternative splicing were significantly differentially regulated in the fetal brain of the short-lived compared to long-lived mice during development from d15 and d17. A significantly reduced number of splice variants was observed on d15 compared to d12 or d17 in a strain-dependent manner. An epigenetic clock analysis of d15 fetal brain identified DNA methylations that were significantly associated with single-nucleotide polymorphic sites between AKR/J and C57BL/6J strains. These methylations were associated with genes that show epigenetic changes in an age-correlated manner in mice. Together, the finding of this study suggest that fetal brain development and longevity are epigenetically linked, supporting the emerging concept of the early-life origin of longevity.

## 1. Introduction

Emerging evidence suggests that early-life developmental processes are linked to the processes that control aging and longevity [1,2,3,4]. Specific molecular and cellular processes are shared between early development and aging [5], which supports the developmental aging (*DevAge*) theory originally proposed by Dilman [6]. Genomics and molecular biology studies have implicated that the *DevAge* link may be widely conserved in mammals [7,8]. Processes associated with development and aging are critically dependent upon common regulatory mechanisms such as nutrient sensing, ribosome biogenesis, and the regulation of alternative splicing by spliceosomes [1,9,10,11]. During mammalian reproduction, development of the fetal organs is dependent on the nutritional supply from the mother to the fetus via the placenta [12]. The fetal brain requires a larger proportion of energy from the maternal resources compared to other organs during fetal development [13]. An imaging analysis has shown specific structural features of the brain that change in a similar pattern during development and aging [3]. Though links between reproduction and longevity are documented [14,15,16,17], the relationship between fetal brain development and longevity remains poorly understood.

Animal models have greatly aided our understanding of the regulation of developmental and longevity processes [18,19]. The murine leukemia viruses (MLVs), which are retroviruses belonging to the gammaretroviral genus, lead to leukemia in these mice. Ecotropic expression of AKV (AK virus) is found in all tissues from birth [20], and leukemia progression occurs in an age-dependent manner in these mice [21,22]. Symptoms of leukemia occur as early as three months of age, and most AKR/J mice (60 to 90%) show severe cancer by the age of 10 months and die within a month or two [20]. AKR/J inbred mice show a nearly 50% reduced longevity compared to that of C57BL6/J mice [23]. Besides variations in longevity among inbred strains of mice [23], the knockout of pro-longevity genes also affects longevity in mice [24]. This is evident from the congenic mouse strain B6.Cg-*Cav1*^tm1Mls^/J generated from the C57BL6/J strain that lacks the Caveolin-1 (*Cav1)* gene. Caveolin-1 is a major structural protein of the flask-shaped lipid pocket of plasma membrane called caveolae. It is abundant in endothelial cells but also present in many other cell types. *Cav1* is a known pro-longevity gene [24]. Additionally, *Cav1* plays important roles in the regulation of angiogenesis [25]. Mice lacking the *Cav1* gene show impaired endothelia function [26]. The ablation of *Cav1* alters the metabolic regulation of the fetal brain [27] and reduce mouse longevity by nearly 50% [28]. *Cav1*-null mice, though viable and fertile, show hyperproliferative and vascular abnormalities [29]. At a young age (3–6 months old), these mice exhibit neuronal aging that resembles that of aged (more than 18 months) mice [30]. *Cav1*-null mice show Alzheimer’s disease (AD)-like symptoms at an early adult life [30]. Increased amyloid β, tau phosphorylation, astrogliosis, and a decreased cerebrovascular volume are observed in the brain of these mice [30]. As *Cav1* plays a key role in modulating β-secretase activity [31], the association of *Cav1* with AD pathologies in these mice is implicated [32]. *Cav1*-null mice are used as models in aging research [33].

In mice, brain development begins around embryonic day 9 (d9) when neural tubes are formed [34]. On d12, the choroid plexus begins to develop along with the medial, lateral, and caudal ganglionic sections, and the hypothalamus becomes bigger in the diencephalon [35]. On d15, all six layers of the cerebral cortex become distinctive, along with a high level of invasion of microglia [36]. On d17, neurogenesis in the fetal brain is most active [36], after which the forebrain, the pituitary gland, and the neuroendocrine structures start growing rapidly.

In the present study, our overall aim is to investigate the genetic regulation of fetal brain development in congenic and inbred mice strains that differ in longevity. Our investigation approach is based on applying integrative approaches to analyze splice variants, epigenetics, and genetic variation to study the genetic regulation of fetal brain development in C57BL6/J, *Cav1*-null, and AKR/J mice at different gestational time points.

## 2. Materials and Methods

### 2.1. Overview of Approach

**Figure 1:**
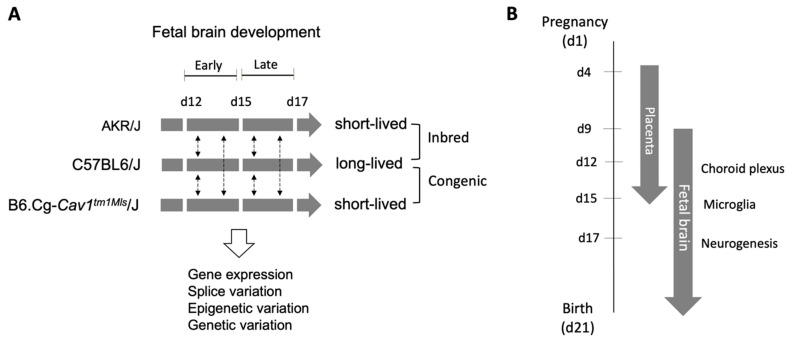
Overview of the study. (**A**). The regulation of fetal brain development was studied in three mice strains on different gestational days (d12, d15, and d17). These mice strains differ in longevity. C57BL/6J and B6.Cg-*Cav1^tm1Mls^*/J (*Cav1*-null) are congenic strains, whereas AKR/J and C57BL/6J are inbred strains. Gene expression changes were investigated in each strain as the brain developed from d12 to d15 (early phase) and from d15 to d17 (late phase), and the differentially expressed genes were compared between strains (dotted vertical arrows). Integrative analyses of gene expression, splice variation, DNA methylation, and genetic variation were performed. (**B**). Key developmental milestones of the fetal brain on d12, d15, and d17 of mouse gestation. The placental development timelines relative to that of fetal brain development are indicated.

The research approach and methodology are schematically illustrated in Figure 1. The developing fetal brain of AKR/J, C57BL/6J, and B6.Cg-*Cav1*^tm1Mls^/J (also known as *Cav1*-null) mice were investigated molecularly on gestational days (d) 12, 15, and 17. These specific time points were chosen for gene expression analysis as mouse fetal brain shows distinct developmental processes at these stages. The relative timelines of placental development are also relevant to brain development because the placenta plays key roles in fetal growth [37,38,39]. On d15, the placenta is fully formed and functional [40], and the fetal brain is at peak in the microglia invasion [36]. In the developmental profiling of gene expression, we referred to the period from d12 to d15 as the “early” phase and from d15 to d17 as the “late” phase of fetal brain development.

### 2.2. Animals and Sample Collection

The inbred mice strains C57BL/6J (strain # 000664), AKR/J (strain # 00064), and B6.Cg-*Cav1^tm1Mls^*/J (strain # 007083) used in this study were purchased from the Jackson Laboratory (Bar Harbor, ME, USA). Approximately 8-week-old mice of these strains were used to establish timed pregnancies separately, as described previously [6]. The vaginal plug was observed to keep a record of the start of pregnancy (day 1). On days (d) 12, 15, and 17, the pregnant mice were euthanized, the fetuses were collected [15] and washed in sterile PBS, and the whole brain was dissected from each fetus [27].

### 2.3. RNA Sequencing

Total RNA was isolated from the dissected fetal brain samples using an AllPrep DNA/RNA Mini Kit (Qiagen, Cat No./ID: 80204, Hilden, Germany) following the manufacturer’s instruction. A total of 27 RNA samples were prepared, which included 3 strains × 3 time points per strain × 3 replicates per time point. The use of three biological replicates per time point followed the recommendation of best practices for RNA-seq analysis [41]. The samples were homogenized with 500 μL RLT buffer (Qiagen, Cat No./ID: 79216). The buffer was freshly supplemented with 5 μL of 2-mercaptoethanol. The homogenate was transferred to a fresh tube and centrifuged for 1 min at ≥8000× *g*. The supernatant (750 μL) was transferred to a fresh tube and mixed with an equal volume of 70% ethanol to precipitate the RNA. RNA was eluted in 30 μL nuclease-free water. The concentration of RNA was determined using a Nanodrop 1000 spectrophotometer (Thermo Fisher Scientific, Saint Louis, MO, USA). The RNA Integrity Number (RIN) was determined using the Agilent 2100 bioanalyzer. The RNA was used for the preparation of sequencing libraries, and the libraries were sequenced (RNA-seq) at the Novogene Cooperation Inc. (Sacramento, CA, USA). Each library was sequenced to 20 million paired-end reads of 150 bases using a NovaSeq 6000 sequencer (Illumina, San Diego, CA, USA).

### 2.4. RNA-Seq Data Analysis

The quality of the raw sequences was checked with *FastQC*. Adaptor trimming was performed using *cutadapt*. The *fastp* tool [42] was used to perform base quality trimming (Phred score > 30) by a sliding-window scan (4 nucleotides). The quality reads were then mapped to the mouse reference genome GRCm39 using the *STAR* aligner [43]. The read count data were analyzed using *edgeR* [44] to determine the significance of the differential expression, as described in our earlier works [27,45,46]. The read count and raw data were submitted to the Gene Expression Omnibus or GEO database (accession number GSE252079).

### 2.5. Detection of Novel and Known Splice Variants in Developing Fetal Brain

We applied the multi-sample 2-pass mapping approach of *STAR* [43] to analyze the splice variants in the fetal brain of the three strains at each developmental time point. The *Ensembl* mouse gene annotation (*Mus_musculus.GRCm39.110.gtf*) was used to count reads mapped to the annotated junction sites of genes in each sample. The junction sites detected from the 1st-pass mapping in each sample were merged with the annotated junctions from the reference genome. The merged junction data were used to perform the 2nd mapping, and the reads mapped to the known or novel junctions were re-counted in each sample. The *sj.out.tab* output files generated from the 2nd mapping were parsed using *awk* commands to filter out junctions with multi-mapped reads or those with less than 20bp overhangs. If a splice junction showed less than 10 mapped reads in all the samples, it was excluded from further analysis. The final list of splice sites detected across the samples was provided as a processed file in the data submission to GEO (accession number GSE252079). When 10 or more reads mapped to the junction site of a gene in a specific strain at a specific time point but not in other strains and developmental time points, that splice junction was counted as a developmental alternative splice variant specific to the strain and time point. The genes associated with the novel splice sites were identified by intersecting the genomic coordinates of the junction sites and the annotated mouse genes (*Mus_musculus.GRCm39.110.gtf*) using *BEDTools* [47].

### 2.6. Analysis of Epigenetic Variation

We profiled the DNA methylation of a total of 2045 CpG (cytosine–guanine) sites associated with the mouse epigenetic clock [48] in the d15 fetal brain from the three mouse strains. DNA from frozen brain samples was purified using the Quick-DNATM Miniprep Plus kit (Cat. No. D4068). Bisulfite DNA conversion was performed from each sample in duplicates using the EZ DNA Methylation-Lightning TM Kit (Cat. No. D5030), followed by enrichment for target loci, library preparation, and sequencing on an Illumina^®^ HiSeq instrument by Zymo Research (Irvine, CA, USA). Data analysis was performed using *Bismark* [49] to extract the methylation sites, and the β-values of the methylation levels for each site were calculated as described previously [50]. Given the heteroscedasticity nature of β-values [50], the β-value of each methylation site was divided by 1 minus β-value and converted to the log_2_ scale. The converted values were subjected to an analysis of variance (ANOVA) to determine the significance of the difference in the methylation level between the strains. ANOVA is a recommend method to access the significance of differences in the methylation level [51]. A one-way ANOVA was performed in R. The hierarchical clustering of methylation sites was performed using the R package *dendextend* (version 1.17.1). The Euclidean distance was used to perform the hierarchical cluster analysis. The difference between the clusters was measured by cophenetic distances and cophenetic correlations [52] using *dendextend*.

### 2.7. Genetic Variant Analysis

We used the single-nucleotide polymorphism (SNP) data (AKR_J.mgp.v5.snps.dbSNP142.vcf) annotated between AKR/J relative to C57BL/6J strain from the Mouse Genomes Project [53]. The SNP data were used to identify genetic variations that were methylated in the fetal brain. In this analysis, we focused on the methylation of the d15 brain samples because our RNA-seq analysis showed that gene regulation between strains was significantly altered in the brain before and after d15. We asked if the differentially methylated (DM) sites in the d15 fetal brain were associated with the known genetic polymorphic sites between the strains in a non-random manner. To answer this question, we calculated the closest distance (in kilobases or kb) between the SNP and DM sites using the *BEDTools closest* function [47]. The distances were divided into different bins (n = 10): 0–0.1, 0.1–1, 1–5, 5–10, 10–50, 50–100, 100–200, 200–400, 400–800, or 800–1000, and the SNPs and DM sites within each bin were counted. For each bin, the count data were converted to 2 × 2 contingency tables that were subjected to Fisher’s exact test to determine if there was a significant SNP-DM association in the individual bins. Fisher’s exact test was appropriate for this analysis as our purpose was to evaluate if methylation variation and genetic variation in a given bin were independent from one another (null hypothesis). Fisher’s tests were performed based on Pearson’s Chi-Square using R.

## 3. Results

### 3.1. Gene Expression Variation in the Fetal Brain of Short- and Long-Lived Mice

**Figure 2:**
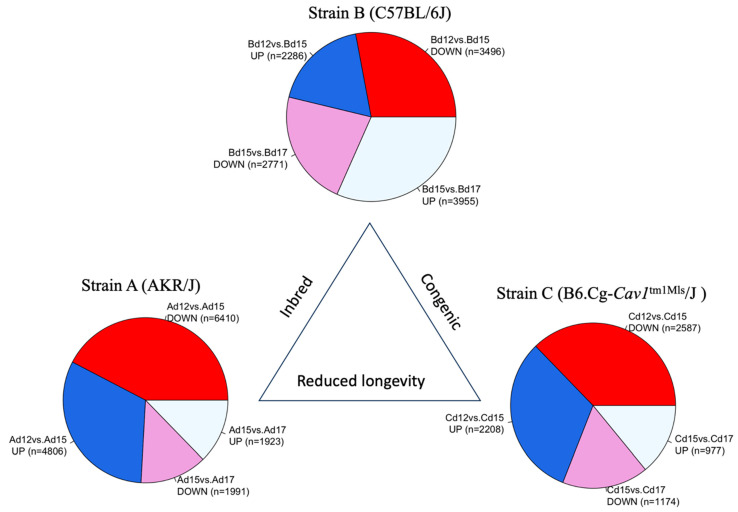
Pie charts show the total number of genes (in parenthesis) that were significantly upregulated (UP) or downregulated (DOWN) between the developmental time points (d12, d15, or d17) in each strain. The strains are shown as A for AKR/J, B for C57BL/6J, and C for B6.Cg-*Cav1^tm1Mls^*/J (*Cav1*-null).

**Figure 3:**
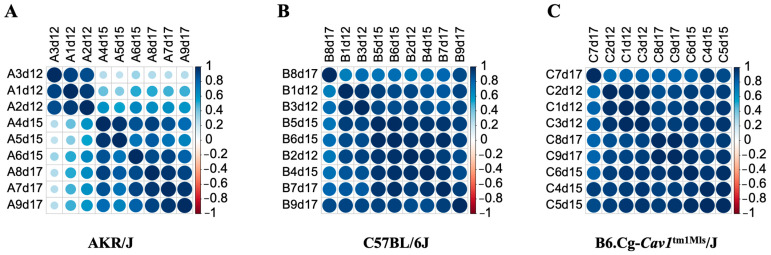
Correlation patterns of gene expression between the developmental times of the fetal brain of strains AKR/J (**A**), C57BL/6J (**B**), and B6.Cg-*Cav1^tm1Mls^*/J (**C**). The scale to the right of each plot shows the color codes for the positive and negative correlations.

We quantified gene expression in fetal brain development of the AKR/J, C57BL/6J, and B6.Cg-*Cav1*^tm1Mls^/J (also known as *Cav1*-null) mice on gestational days (d) 12, 15, and 17. The gene expression data (Supplementary Table S1) were analyzed in a pair-wise manner between strains during the two developmental phases—d12 versus d15 and d15 versus d17. The differential gene expression analysis [44] showed that specific genes were significantly (false discovery rate < 0.05) changed in their expression during the two developmental phases of the fetal brain (Supplementary Figure S1). The list of the differentially expressed (DE) genes is provided in Supplementary Table S2. A greater number of genes were differentially expressed in the early phase compared to the late phase of brain development in AKR/J and *Cav1*-null mice (short-lived mice) (Figure 2). In contrast, a greater number of genes were differentially expressed in the late phase compared to the early phase of brain development in the C57BL/6J (long-lived) mice. Cramer’s V [54] was greater than 0.2 when the number of DE genes was compared in a Chi-Square test either between the AKR/J and C57BL/6J (Chi-Square = 2355.2, degree of freedom or df = 3, *p* < 0.0001, and Cramer’s V = 0.291) or between the *Cav1*-null and C57BL/6J (Chi-Square = 1079.9, df = 3, *p* < 0.0001, and Cramer’s V = 0.235). But, when the DE gene counts were compared between the AKR/J and *Cav1*-null mice (both short-lived), Cramer’s V was less than 0.061 (Chi-Square = 83.3, df = 3, *p* < 0.0001). Of note, Cramer’s V is a measure, varying from 0 to 1, representing how strongly two variables are associated with one another (0 means no association, and 1 means complete association). Our analysis suggested that the observed changes in gene expression were significantly associated with the developmental timing of the fetal brain in a strain-dependent manner. The gene expression pattern was significantly different on d12 compared to d15 or d17 in the AKR/J strain, while this pattern was not observed in the C57BL/6J or *Cav1*-null strain (Figure 3).

**Figure 4:**
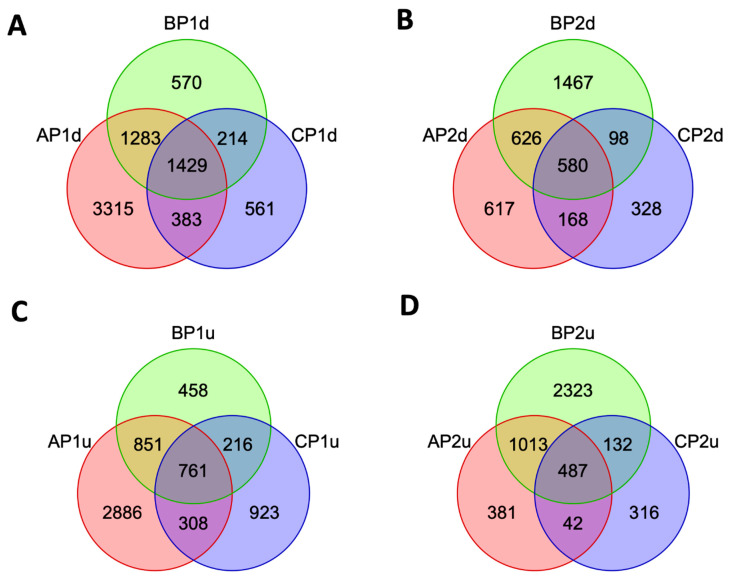
Venn diagrams showing the number of genes significantly downregulated (d) or upregulated (u) during fetal brain development. The strains are shown as A for AKR/J, B for C57BL/6J, and C for B6.Cg-*Cav1^tm1Mls^*/J (*Cav1*-null). The downregulated genes are shown in (**A**,**B**). The upregulated genes are shown in (**C**,**D**). P1 represents the early phase (from d12 to d15) and P2 represents the late phase (from d15 to d17) of brain development.

The number of genes upregulated or downregulated in the fetal brain varied in a strain-specific manner (Figure 4). The number of strain-specific downregulated genes is shown in Figure 4A,B. The number of strain-specific upregulated genes is shown in Figure 4C,D. In these Venn diagrams, the strains are shown as A for AKR/J, B for C57BL/6J, and C for B6.Cg-*Cav1^tm1Mls^*/J (*Cav1*-null) mice. The figure showed that a relatively greater number of genes were downregulated in strain A compared to strain B or C during the early phase (P1) of brain development. When the brain developed to phase 2 (P2), a greater number of genes were downregulated in strain B compared to strain A or C. A similar pattern was also observed among the strain-specific upregulated genes.

### 3.2. Functional Annotation of the Differentially Expressed Genes in the Fetal Brain

The gene groups shown in Figure 4 were subjected to Gene Ontology (GO) enrichment analysis [55]. The analysis showed that the genes commonly downregulated in the AKR/J and C57BL/6J brains were related to the nervous system development, neurogenesis, and system development functions (Supplementary Table S3). These functions were not enriched with genes commonly downregulated in *Cav1*-null and C57BL/6J mice. On the other hand, the genes commonly upregulated in the early phase of brain development in AKR/J and C57BL/6J mice were enriched with ribosome biogenesis processes such as rRNA processing, rRNA metabolic process, ribonucleoprotein complex assembly, ribonucleoprotein complex-subunit organization, ribosomal large-subunit biogenesis, ribosome assembly, and ribosomal small-subunit biogenesis (Supplementary Table S4). The genes upregulated in the *Cav1*-null and C57BL/6J strains during the early phase of brain development were associated with functions related to brain development such as cell proliferation in the hindbrain, brain development, cell differentiation in the hindbrain, hindbrain development, hindbrain morphogenesis, and forebrain generation of neurons. The genes upregulated in the same strains during the late phase of brain development were associated with spliceosome functions such as spliceosomal tri-snRNP complex assembly, spliceosomal snRNP assembly, spliceosomal tri-snRNP complex, and spliceosomal snRNP complex. In addition to the GO enrichment analysis, a pathway enrichment analysis showed that specific pathways, including those related to Alzheimer disease, carcinoma, leukemia, and viral and parasitic infections, were significantly enriched by the DE genes (Supplementary Table S5).

### 3.3. Alternative Splice Variants in the Fetal Brain of Short- versus Long-Lived Mice

**Figure 5:**
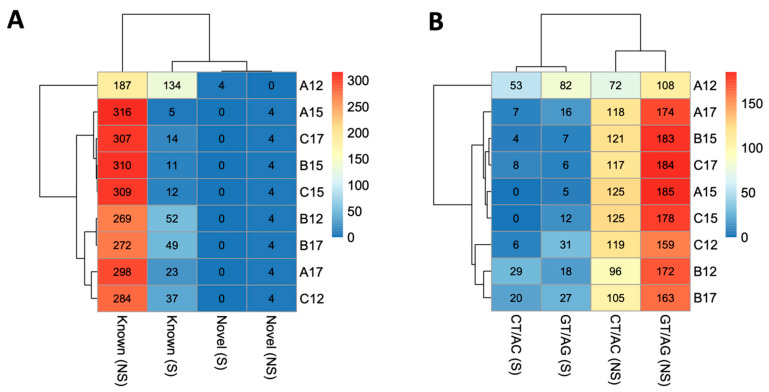
(**A**). Heatmap showing variations in the number of known or novel splice variants in the fetal brain of the three mouse strains at different development stages. (**B**). Heatmap showing variations in the number of the most abundant junction motifs (GT/AG and CT/AC) associated with the splice variants in the fetal brain. In both plots, the mouse strains are shown as A for AKR/J, B for C57BL/6J, and C for B6.Cg-*Cav1^tm1Mls^*/J (*Cav1*-null), and the developmental time points are shown as 12, 15, or 17 gestational days. The time-specific splice sites are shown as S in the parentheses. The non-specific splice sites are shown as NS. The scale on the right shows the color codes for the number of splice variants.

**Figure 6:**
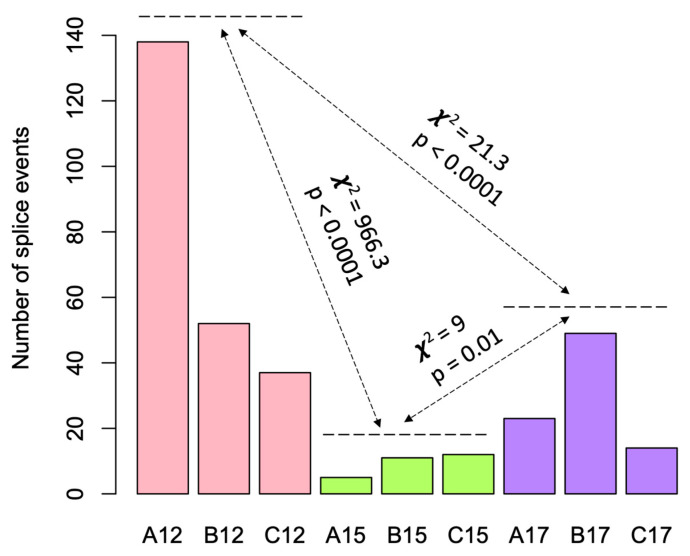
A significantly greater number of splice variants in the fetal brain at either the early (d12) or late (d17) time points compared to the mid (d15) time point. The bar plot shows the number of splice variants that were identified in each strain in a developmental time-specific manner. The strains are shown as A for AKR/J, B for C57BL/6J, and C for B6.Cg-*Cav1^tm1Mls^*/J (*Cav1*-null), and the developmental time points are shown as 12, 15, or 17 gestational days. On d15, the fetal brain showed fewer splice variants compared to either d12 or d17 across the three strains. The Chi-Square and *p*-values are shown for d12 vs. d15 and d15 vs. d17 comparisons for the number of splice variants.

As alternative splice variants play vital roles during brain development [56], we performed a splice variant analysis in the developing fetal brain of the three mouse strains. A total of 130,424 splice variants were identified in the fetal brain among the three strains (data available in GEO, accession # GSE252079). Based on the total number of the known protein-coding genes (n = 21,684) in the mouse genome, this accounted for an average of six splice variants per gene in the fetal brain. Specific splice variants (n = 378) were identified in the fetal brain in a developmental time-specific manner (Supplementary Table S6), the majority (46%, n = 174) of which were observed in the AKR/J strain on d12. Besides these time-specific (S) splice variants, we also observed common splice variants at more than one time point, referred to as non-specific (NS) splice variants (Figure 5A). The heatmap in Figure 5A shows the number of known or novel splice variants that are either specific (S) or non-specific (NS) to the developmental times of the fetal brain. Figure 5B shows the number of the two most abundant motif sequences of the splice junction sites (GT/AG and CT/AC) [57] associated with the S and NS splice variants. The S variants were significantly reduced on d15 compared to d12 or d17 (Figure 6). The GO enrichment analysis showed that the genes associated with the splice variants were significantly associated with functions related to the cell cycle and its regulation, DNA damage and repair, chromosome segregation, spindle organization, and the regulation of cytokinesis (Supplementary Table S7).

### 3.4. DNA Methylation Changes in the Fetal Brain between Strains

**Figure 7:**
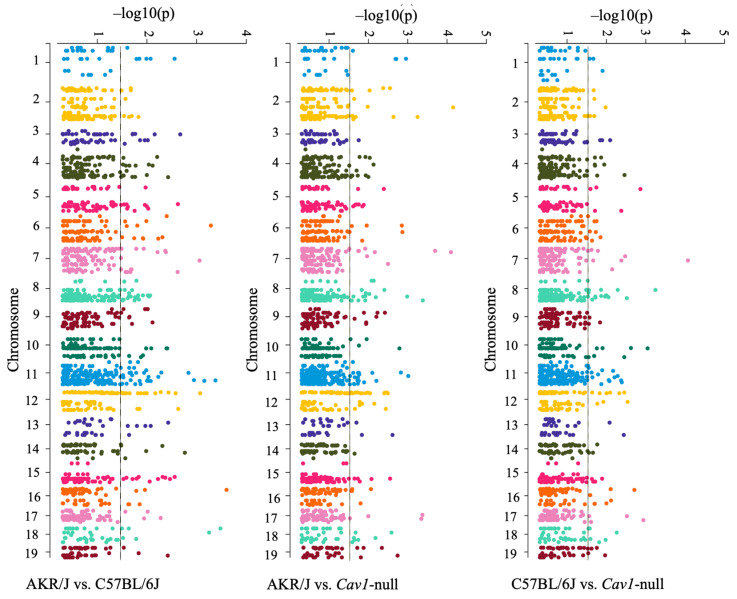
Manhattan plots showing the chromosome-level distribution of the CpG sites differentially methylated in the d15 fetal brain between the strains. The strains compared are shown below the plots. Each dot represents a methylation site. The dots are color-coded based on the chromosomes. The vertical lines show the significance level of the *p*-value (<0.05). Dots to the right of these lines are significantly different in the methylation level between the strains.

**Figure 8:**
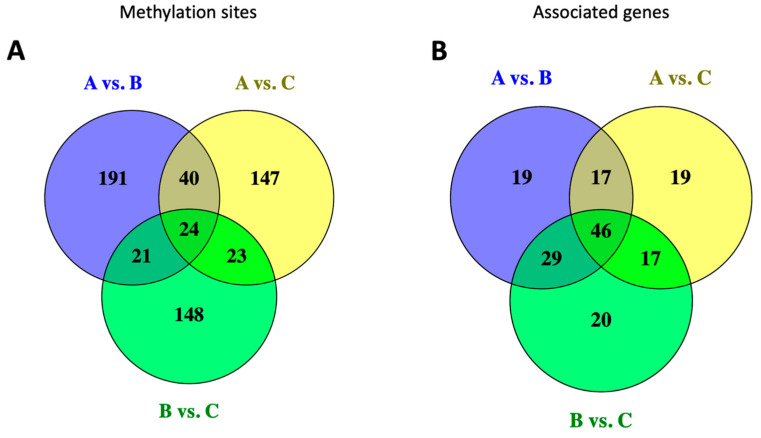
Venn diagrams showing the number of differentially methylated sites in the d15 fetal brain between the strains. (**A**). Number of methylation sites. (**B**). Number of genes associated with these methylations. The strains are shown as A for AKR/J, B for C57BL/6J, and C for B6.Cg-*Cav1^tm1Mls^*/J (*Cav1*-null).

**Figure 9:**
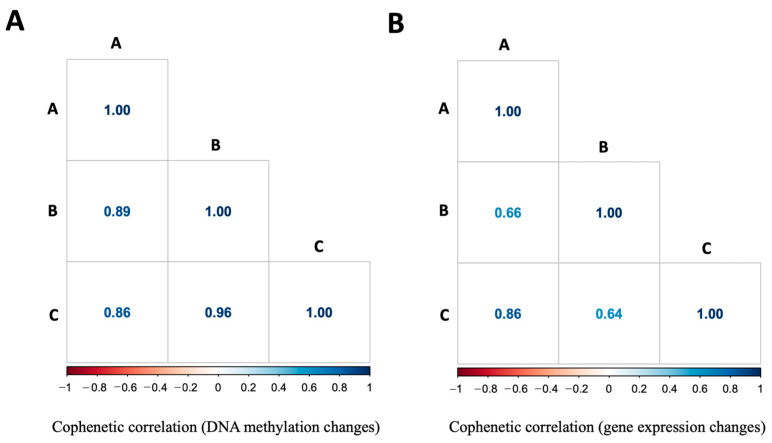
Comparison of the cophenetic correlations of methylation changes (**A**) and gene expression changes (**B**) in the d15 fetal brain among the three strains. The strains are shown as A for AKR/J, B for C57BL/6J, and C for B6.Cg-*Cav1^tm1Mls^*/J (*Cav1*-null). The scales below the plots show the correlation levels.

Next, we asked if DNA methylation occurred differentially in the fetal brain between the short- and long-lived mice. To test this hypothesis, we profiled the methylation level of the CpG sites (n = 2045) associated with the mouse epigenetic clock [48,58] in the d15 fetal brains of the AKR/J, C57BL/6J, and *Cav1*-null mice. The methylation data are provided in Supplementary Table S8. Our data analysis identified a total of 594 CpG sites that were significantly differentially methylated in the fetal brain between the strains (Figure 7). Each dot in the Manhattan plots shown in Figure 7 represents a CpG methylation, and those located outside of the vertical line represented the DM sites that significantly (*p*-value < 0.05) differed in the methylation level between the strains. A relatively greater number of DM sites were identified between AKR/J and C57BL/6J compared to those between *Cav1*-null and C57BL/6J mice (Figure 8A). A total of 24 sites were commonly altered in the fetal brain among all three strains. The DM sites were associated with different genes (Figure 8B). We investigated the relationships between methylation changes and expression changes in the associated genes among the three strains. The cophenetic correlation measure was determined [52] from the hierarchical cluster patterns of the methylation and gene expression data. The analysis showed that the changes in the methylation level were correlated among each other in a differential manner in the short- versus long-lived mice (0.96 for C57BL/6J vs. *Cav1*-null, and 0.89 for C57BL/6J vs. AKR/J) (Figure 9A). But the expression changes of the genes associated with these methylations showed a reduced level of correlation between the strains. The correlations were 0.64 for C57BL/6J vs. *Cav1*-null and 0.66 for C57BL/6J vs. AKR/J mice (Figure 9B).

### 3.5. Influence of Genetic Variation on the Regulation of Fetal Brain Development

We further investigated if the epigenetic variation of the fetal brain was associated with the known genetic polymorphisms between the AKR/J and C57BL/6J mice. To test this hypothesis, we analyzed the SNP data annotated in the AKR/J mice relative to the C57BL/6J strain from the Mouse Genomes Project [53]. In this analysis (see Methods for details), we determined the significance of associations between methylation variation and genetic variations (SNPs) in the fetal brain. The results of this analysis showed a non-random association between genetic variation and the observed epigenetic variations in the fetal brain (Table 1). A significant association was observed between the DM and SNP pairs when they were localized within a 1 kb distance from one another. Methylations that were further away (>1 kb) from the SNPs did not show this association (Table 1). The list of SNPs found in close proximity (within 1 kb) with the differential methylations in the fetal brain is provided in Supplementary Table S9. A representative genome browser view (IGV: Integrative Genomics Viewer) showing the co-localization SNP and methylation sites is shown in the Supplementary Figure S2. These genetic–epigenetic associations were identified in specific genes such as *C1qtnf1*, *Cadm1*, *Gas7*, *Gga1*, *Gm2093, Il17rd*, *Lncppara*, *Map10*, *Mpp5*, *Rbpms2*, *Rusc2*, *Sh3pxd2a*, *Zfhx3*, and *Zscan2*, which are known to be methylated in an age-correlated manner in mice [48,58].

## 4. Discussion

This study was initiated by investigating the genetic regulation of fetal brain development in short- and long-lived mice. The median number of days during which AKR/J mice can survive varies from 251 to 288 days, while that of C57BL/6J mice varies from 866 to 901 days [23]. On the other hand, the ablation of *Cav1* causes a nearly 50% reduced longevity in mice. These short-lived (AKR/J or *Cav1*-null) mice show pathological conditions later in life, unlike the C57BL/6J mice. The AKR/J mice develop leukemia [20], while the *Cav1*-null mice show neurodegeneration and premature aging in an early adult life [32]. Though both the AKR/J and *Cav1*-null strains show reduced longevity relative to the C57BL/6J strain, the genetic makeups of these two strains differ from one another. *Cav1*-null mice are congenic to the C57BL/6J strain. These mice were generated by breeding the *Cav1^tm1Mls^*/J (donor) mice with the C57BL/6J (receiver) strain and then through repeated backcrossing the F1s to the C57BL6/J strain. On the other hand, AKR/J and C57BL/6J are inbred strains. Genome sequencing showed extensive genetic variations in AKR/J [53] relative to the C57BL/6J strain. However, in both AKR/J and *Cav1*-null mice, we observed a greater number of genes being differentially expressed during the early phase (d12 to d15) compared to the late phase (d15 to d17). In contrast, a greater number of genes were differentially regulated in the fetal brain of C57BL/6J mice compared to AKR/J and *Cav1*-null mice during the late phase of brain development (Figure 2).

A common theme emerged from the GO enrichment analysis, which showed that genes related to specific developmental and aging processes were differentially regulated in the fetal brain (Supplementary Table S4). We observed that genes significantly enriched with ribosome biogenesis were commonly upregulated during the early phase but not during the late phase of brain development both in AKR/J and C57BL/6J. When we compared the genes commonly upregulated in the brains of *Cav1*-null and C57BL/6J mice, a different pattern emerged. In this case, the genes related to developmental processes were upregulated in both strains during the early phase whereas the genes related to the regulation of alternative splicing were upregulated during the late phase of brain development. These results suggested that the genes related to a combination of developmental and aging processes and the regulation of alternative splicing processes were tightly controlled during fetal brain development. While AKR/J mice show the ecotropic expression of endogenous retroviral genes (AK virus) [59], *Cav1*-null mice show metabolic and cellular abnormalities in the fetal brain [27]. Despite these developmental abnormalities, *Cav1*-null mice are not embryonic-lethal, suggesting that these mice might have developed adaptive mechanisms to counter these early-life developmental abnormalities.

We observed a significant enrichment of ribosome biogenesis and spliceosomal functions that were activated only in the fetal brain of the short-lived mice. Ribosome biogenesis is a well-known pathway that regulates longevity [10]. Similarly, spliceosomes also play a key role in producing the alternative splicing of genes associated with aging and longevity [60]. Furthermore, our pathway enrichment analysis (Supplementary Table S5) showed that the pathways related to leukemia and Alzheimer disease were significantly enriched by genes differentially expressed in the fetal brain of the short-lived mice (Supplementary Table S5), suggesting that these pathways were regulated in the developing brain of the AKR/J and *Cav1*-null mice which show leukemia and Alzheimer disease, respectively, later in life.

The results of the present study showed that alternative splice variants played a role in the regulation of fetal brain development. The S variants (developmental time-specific splice variants) were significantly reduced in their number on d15 compared to those either on d12 or d17. The AKR/J mice showed the greatest number of S variants in the fetal brain compared to those in either the C57BL/6J or *Cav1*-null mice on d12. The greatest number of DE genes was also observed in the AKR/J mice at this time point, suggesting a role of splice variants in the differential regulation of cognate genes in the fetal brain, supporting the role of splice variants in the regulation of gene expression [61]. We further observed that the genes associated with alternative splicing in the fetal brain were significantly enriched with processes such as DNA repair, recombinational repair, double-strand break repair via homologous recombination, chromosome segregation, and spindle organization (Supplementary Table S7). These functions are related to adaptive responses to aging and disease-related stress conditions [62,63,64].

The key finding of this study is that differential gene expression in the fetal brain is epigenetically regulated. DNA methylation in the fetal brain showed a relatively reduced similarity in the cluster patterns between the AKR/J and *Cav1*-null strains compared to that between the *Cav1*-null and C57BL/6J or AKR/J and C57BL/6J mice. However, the gene expression pattern showed an opposite pattern. These opposite relationships between gene expression and DNA methylation changes were consistent with known effects of DNA methylation on gene expression [65], which were also evident in our data (Figure 9). The results of this study further showed evidence for a non-random association between genetic variations with the observed epigenetic variations in the fetal brain. We identified DM sites that were enriched within 1 kb of SNPs identified in AKR/J compared to the C57BL/6J strain. These methylations were associated with the genes that regulated the epigenetic clock of aging in mice, supporting the idea that brain development and longevity are epigenetically linked [2,46,66,67].

The connection between brain development and longevity may be linked to energy consumption by the brain during fetal development [68]. An earlier study suggested that reproduction and life-span are associated with common regulatory processes that center around nutrient utilization and signaling [19]. During reproduction, more than 20% of the available energy is used by the brain alone, while this organ represents only ~2% of the total body mass [69]. In fact, the fetal brain consumes an amount of energy that is equivalent to the energy consumed by the adult brain [70]. Due to the large allocation of maternal energy to the developing brain which is supplied to the fetus via the placenta, the regulation of placental functions and brain development are intricately related [71]. Influences of the placenta on the development of the fetal brain are well documented [37,72,73,74,75]. The regulation of the fetal brain and placental axis is vulnerable to maternal nutrition during pregnancy [76,77,78]. The epigenome of the placenta influences fetal programming for brain aging in mice [45,46]. Recently, we showed that the conditional knockout of the RE1 silencing transcription factor (REST), a regulator of neurogenesis and longevity, in the placenta significantly altered gene expression of the offspring brain in adult life [45]. REST plays key roles to control the maintenance of pluripotency and the self-renewal of stem cells in the developing fetus [79] and regulates processes associated with longevity [80], suggesting that REST can be a potential regulator of functional links between fetal brain development and longevity.

The present study also has certain limitations. This study does not address how placental development and/or function are regulated in short-lived mice compared to long-lived mice. It also does not address if the genetic and epigenetic association is transgenerational and if epigenetic inheritance can be a driving force for the evolution of differential longevities between strains [81]. It is also unknown if energy allocation by the placenta plays a role in the trade-off between early-life development and longevity [82]. These are important issues regarding the ongoing efforts by different biotech companies that are leveraging placenta-derived mesenchymal stem cell therapy to develop anti-aging intervention treatments to extend human longevity [83,84,85].

Despite these limitations, the findings of the present study have significant broader implications. Our study suggests a role of epigenetic alteration in SNP loci in the fetal brain of mice that develop leukemia later in life. Epigenetics plays a significant role in the regulation of the programmed senescence of cells [86]. In humans, programmed cellular senescence contributes to neurodegeneration and debilitating brain diseases [87]. The association between leukemia and cognitive aging is also a major health issue among people living with blood cancer [88]. A cross-sectional study has shown a functional decline in cognition among people with chronic lymphatic leukemia [89]. Though correspondences in DNA methylation between the blood and the brain are well documented [90,91,92,93,94,95], whether leukemia and brain aging are epigenetically linked to blood DNA and the programmed senescence of brain cells begs further investigation. Besides DNA methylation, our findings also suggest that alternative splicing plays a role in the differential regulation of brain development between short- and long-lived mice. While alternative splicing is well documented in the aging brain [96,97,98], our data suggest that the alternative splicing of brain genes may influence the molecular links between early development and aging. In conclusion, the findings of this study collectively suggest that molecular changes, including epigenetic alterations, in the developing fetal brain are functionally linked to the variation in longevity in mice.

## Figures and Tables

**Table 1:** Significance of the association between the number of SNP and DM sites identified in each SNP-DM distance bin.

SNP-DM Distance Bins	SNP Count (Within Bin)	DM Count (Within Bin)	SNP Count (Not in the Bin)	DM Count (Not in the Bin)	Odds Ratio	*p*-Value
0–0.1 kb	38	170	277	1787	1.4418	0.03737
0.1–1 kb	62	302	254	1655	1.3375	0.03802
1–5 kb	55	357	252	1600	0.9782	0.58150
5–10 kb	23	162	282	1795	0.9038	0.70291
10–50 kb	87	609	217	1348	0.8875	0.82764
50–100 kb	27	177	276	1780	0.9838	0.56439
100–200 kb	20	144	283	1813	0.8898	0.71749
200–400 kb	4	20	299	1937	1.2955	0.40478
400–800 kb	2	11	301	1946	1.1754	0.53688
800–1000 kb	1	5	302	1952	1.2926	0.57884

## Data Availability

All the raw and processed data from the RNA-seq analysis were submitted to the GEO database under accession number GSE252079. The methylation data are provided as a Supplemental File alongside this paper.

## Supplementary Materials

The following supporting information can be downloaded at https://www.mdpi.com/article/10.3390/genes15050604/s1; Table S1: Gene expression data (read counts) of fetal brain samples of the three strains. The Ensembl ID of genes are listed. The strains are abbreviated as A for AKR/J, B for C57BL/6J and C for B6.Cg-*Cav1tm1Mls*/J, Table S2: Differential gene expression analysis results. The abbreviations used are logFC: log fold changes of gene expression, logCPM: log counts per million of reads; LR: likelihood ratio; *p* Value: raw *p*-value; Table S3: The top three GO terms associated with each gene group, Table S4: List of all the GO terms of biological, molecular and cellular functions significantly over-represented with each gene group, Table S5: Significant enrichment of pathways by the DE gene groups, Table S6: List of splice variants produced in the fetal brain at specific developmental time points in the mouse strains. The number of the mapped reads to the splice junction sites in each sample is shown. If a junction site is novel, it is indicated so. The motif types of the junction sites are also shown. The strains are abbreviated as A for AKR/J, B for C57BL/6J and C for B6.Cg-*Cav1tm1Mls*/J (*Cav1*-null). The gestation days of brain samples are shown d12, d15 and d17, Table S7: List of GO terms associated with the alternative spliced genes in the fetal brain, Table S8: List of methylation sites and the beta-values of methylation level in the d15 fetal brain (in two replicates) of the strains A (AKR/J), B (C57BL/6J) and C (*Cav1*-null), Table S9: The list of SNPs found in close proximity (within 1 kb) with the CpG sites differentially methylated in d15 fetal brain between the strains, Figure S1: Volcano plots showing significantly upregulated genes (orange) or downregulated genes (green) between developmental times in each strain. The title on the top of each plot shows the time points (d12, d15 or d17) in the strain (A for AKR/J, B for C57BL/6J and C for B6.Cg-*Cav1tm1Mls*/J (*Cav1*-null) mice. The *x*-axis represents the fold changes in gene expression in log scale, and *y*-axis represent the significance of differential expression (false discovery rate) in log scale, Figure S2: Genome browser view of SNPs identified in close proximity (<100 bp) of CpG sites differentially methylated between the strains. The strains are shown as A for AKR/J, B for C57BL/6J and C for B6.Cg-*Cav1tm1Mls*/J (*Cav1*-null). The methylation positions are shown as chromosome coordinate below the genes. The SNP IDs are shown below the methylation sites. The strains compared are shown on the left of the plots.

## References

[1] Dalgaard C.-J., Hansen C.W., Strulik H. (2021). Fetal Origins-A Life Cycle Model of Health and Aging from Conception to Death. Health Econ..

[2] Hadad N., Masser D.R., Blanco-Berdugo L., Stanford D.R., Freeman W.M. (2019). Early-Life DNA Methylation Profiles Are Indicative of Age-Related Transcriptome Changes. Epigenetics Chromatin.

[3] Tamnes C.K., Walhovd K.B., Dale A.M., Østby Y., Grydeland H., Richardson G., Westlye L.T., Roddey J.C., Hagler D.J., Due-Tønnessen P. (2013). Brain Development and Aging: Overlapping and Unique Patterns of Change. Neuroimage.

[4] Vaiserman A. (2019). Early Life Origins of Ageing and Longevity.

[5] Marchionni S., Sell C., Lorenzini A. (2020). Development and Longevity: Cellular and Molecular Determinants—A Mini-Review. Gerontology.

[6] Dilman V.M. (1971). Age-Associated Elevation of Hypothalamic, Threshold to Feedback Control, and Its Role in Development, Ageine, and Disease. Lancet.

[7] Feltes B.C., de Faria Poloni J., Bonatto D. (2015). Development and Aging: Two Opposite but Complementary Phenomena. Interdiscip. Top. Gerontol..

[8] de Magalhães J.P., Church G.M. (2005). Genomes Optimize Reproduction: Aging as a Consequence of the Developmental Program. Physiology.

[9] Bartke A. (2015). Early Life Events Can Shape Aging and Longevity. Curr. Aging Sci..

[10] MacInnes A.W. (2016). The Role of the Ribosome in the Regulation of Longevity and Lifespan Extension. Wiley Interdiscip. Rev. RNA.

[11] Pitale S., Sahasrabuddhe A. (2011). Fetal Origin of Vascular Aging. Indian. J. Endocrinol. Metab..

[12] Cheatham C.L. (2019). Nutritional Factors in Fetal and Infant Brain Development. Ann. Nutr. Metab..

[13] Steiner P. (2019). Brain Fuel Utilization in the Developing Brain. Ann. Nutr. Metab..

[14] Aguilaniu H. (2015). The Mysterious Relationship between Reproduction and Longevity. Worm.

[15] Antebi A. (2013). Regulation of Longevity by the Reproductive System. Exp. Gerontol..

[16] Barker D.J.P., Osmond C., Thornburg K.L., Kajantie E., Eriksson J.G. (2011). The Lifespan of Men and the Shape of Their Placental Surface at Birth. Placenta.

[17] Templeman N.M., Murphy C.T. (2018). Regulation of Reproduction and Longevity by Nutrient-Sensing Pathways. J. Cell Biol..

[18] Mitchell S.J., Scheibye-Knudsen M., Longo D.L., de Cabo R. (2015). Animal Models of Aging Research: Implications for Human Aging and Age-Related Diseases. Annu. Rev. Anim. Biosci..

[19] Phillips N.L.H., Roth T.L. (2019). Animal Models and Their Contribution to Our Understanding of the Relationship Between Environments, Epigenetic Modifications, and Behavior. Genes.

[20] Herr W., Gilbert W. (1982). Germ-Line MuLV Reintegrations in AKR/J Mice. Nature.

[21] Hays E.F., Beck W.S. (1958). The Development of Leukemia and Other Neoplasms in Mice Receiving Cell-Free Extracts from a High-Leukemia (AKR) Strain. Cancer Res..

[22] Langlois M.-A., Kemmerich K., Rada C., Neuberger M.S. (2009). The AKV Murine Leukemia Virus Is Restricted and Hypermutated by Mouse APOBEC3. J. Virol..

[23] Yuan R., Tsaih S.-W., Petkova S.B., de Evsikova C.M., Xing S., Marion M.A., Bogue M.A., Mills K.D., Peters L.L., Bult C.J. (2009). Aging in Inbred Strains of Mice: Study Design and Interim Report on Median Lifespans and Circulating IGF1 Levels. Aging Cell.

[24] Folgueras A.R., Freitas-Rodríguez S., Velasco G., López-Otín C. (2018). Mouse Models to Disentangle the Hallmarks of Human Aging. Circ. Res..

[25] Chang S.-H., Feng D., Nagy J.A., Sciuto T.E., Dvorak A.M., Dvorak H.F. (2009). Vascular Permeability and Pathological Angiogenesis in Caveolin-1-Null Mice. Am. J. Pathol..

[26] Frank P.G., Woodman S.E., Park D.S., Lisanti M.P. (2003). Caveolin, Caveolae, and Endothelial Cell Function. Arterioscler. Thromb. Vasc. Biol..

[27] Islam M., Behura S.K. (2023). Role of Caveolin-1 in Metabolic Programming of Fetal Brain. iScience.

[28] Park D.S., Cohen A.W., Frank P.G., Razani B., Lee H., Williams T.M., Chandra M., Shirani J., De Souza A.P., Tang B. (2003). Caveolin-1 Null (−/−) Mice Show Dramatic Reductions in Life Span. Biochemistry.

[29] Razani B., Engelman J.A., Wang X.B., Schubert W., Zhang X.L., Marks C.B., Macaluso F., Russell R.G., Li M., Pestell R.G. (2001). Caveolin-1 Null Mice Are Viable but Show Evidence of Hyperproliferative and Vascular Abnormalities. J. Biol. Chem..

[30] Head B.P., Peart J.N., Panneerselvam M., Yokoyama T., Pearn M.L., Niesman I.R., Bonds J.A., Schilling J.M., Miyanohara A., Headrick J. (2010). Loss of Caveolin-1 Accelerates Neurodegeneration and Aging. PLoS ONE.

[31] Hattori C., Asai M., Onishi H., Sasagawa N., Hashimoto Y., Saido T.C., Maruyama K., Mizutani S., Ishiura S. (2006). BACE1 Interacts with Lipid Raft Proteins. J. Neurosci. Res..

[32] Wang F., Cao Y., Ma L., Pei H., Rausch W.D., Li H. (2018). Dysfunction of Cerebrovascular Endothelial Cells: Prelude to Vascular Dementia. Front. Aging Neurosci..

[33] Zou H., Stoppani E., Volonte D., Galbiati F. (2011). Caveolin-1, Cellular Senescence and Age-Related Diseases. Mech. Ageing Dev..

[34] Greene N.D.E., Copp A.J. (2014). Neural Tube Defects. Annu. Rev. Neurosci..

[35] Chen V.S., Morrison J.P., Southwell M.F., Foley J.F., Bolon B., Elmore S.A. (2017). Histology Atlas of the Developing Prenatal and Postnatal Mouse Central Nervous System, with Emphasis on Prenatal Days E7.5 to E18.5. Toxicol. Pathol..

[36] Reemst K., Noctor S.C., Lucassen P.J., Hol E.M. (2016). The Indispensable Roles of Microglia and Astrocytes during Brain Development. Front. Hum. Neurosci..

[37] Behura S.K., Dhakal P., Kelleher A.M., Balboula A., Patterson A., Spencer T.E. (2019). The Brain-Placental Axis: Therapeutic and Pharmacological Relevancy to Pregnancy. Pharmacol. Res..

[38] Broad K.D., Keverne E.B. (2011). Placental Protection of the Fetal Brain during Short-Term Food Deprivation. Proc. Natl. Acad. Sci. USA.

[39] Woods L., Perez-Garcia V., Hemberger M. (2018). Regulation of Placental Development and Its Impact on Fetal Growth—New Insights From Mouse Models. Front. Endocrinol..

[40] Watson E.D., Cross J.C. (2005). Development of Structures and Transport Functions in the Mouse Placenta. Physiology.

[41] Conesa A., Madrigal P., Tarazona S., Gomez-Cabrero D., Cervera A., McPherson A., Szcześniak M.W., Gaffney D.J., Elo L.L., Zhang X. (2016). A Survey of Best Practices for RNA-Seq Data Analysis. Genome Biol..

[42] Chen S., Zhou Y., Chen Y., Gu J. (2018). Fastp: An Ultra-Fast All-in-One FASTQ Preprocessor. Bioinformatics.

[43] Dobin A., Davis C.A., Schlesinger F., Drenkow J., Zaleski C., Jha S., Batut P., Chaisson M., Gingeras T.R. (2013). STAR: Ultrafast Universal RNA-Seq Aligner. Bioinformatics.

[44] Robinson M.D., McCarthy D.J., Smyth G.K. (2010). edgeR: A Bioconductor Package for Differential Expression Analysis of Digital Gene Expression Data. Bioinformatics.

[45] Islam M., Samal A., Davis D.J., Behura S.K. (2024). Ablation of Placental REST Deregulates Fetal Brain Metabolism and Impacts Gene Expression of the Offspring Brain at the Postnatal and Adult Stages. FASEB J..

[46] Islam M., Strawn M., Behura S.K. (2022). Fetal Origin of Sex-Bias Brain Aging. FASEB J..

[47] Quinlan A.R., Hall I.M. (2010). BEDTools: A Flexible Suite of Utilities for Comparing Genomic Features. Bioinformatics.

[48] Stubbs T.M., Bonder M.J., Stark A.-K., Krueger F., von Meyenn F., Stegle O., Reik W., BI Ageing Clock Team (2017). Multi-Tissue DNA Methylation Age Predictor in Mouse. Genome Biol..

[49] Krueger F., Andrews S.R. (2011). Bismark: A Flexible Aligner and Methylation Caller for Bisulfite-Seq Applications. Bioinformatics.

[50] Du P., Zhang X., Huang C.-C., Jafari N., Kibbe W.A., Hou L., Lin S.M. (2010). Comparison of Beta-Value and M-Value Methods for Quantifying Methylation Levels by Microarray Analysis. BMC Bioinform..

[51] Shafi A., Mitrea C., Nguyen T., Draghici S. (2017). A Survey of the Approaches for Identifying Differential Methylation Using Bisulfite Sequencing Data. Brief. Bioinform..

[52] Galili T. (2015). Dendextend: An R Package for Visualizing, Adjusting and Comparing Trees of Hierarchical Clustering. Bioinformatics.

[53] Lilue J., Doran A.G., Fiddes I.T., Abrudan M., Armstrong J., Bennett R., Chow W., Collins J., Collins S., Czechanski A. (2018). Sixteen Diverse Laboratory Mouse Reference Genomes Define Strain-Specific Haplotypes and Novel Functional Loci. Nat. Genet..

[54] McHugh M.L. (2013). The Chi-Square Test of Independence. Biochem. Med..

[55] Mi H., Muruganujan A., Casagrande J.T., Thomas P.D. (2013). Large-Scale Gene Function Analysis with the PANTHER Classification System. Nat. Protoc..

[56] Su C.-H., Dhananjaya D., Tarn W.-Y. (2018). Alternative Splicing in Neurogenesis and Brain Development. Front. Mol. Biosci..

[57] Burset M., Seledtsov I.A., Solovyev V.V. (2000). Analysis of Canonical and Non-Canonical Splice Sites in Mammalian Genomes. Nucleic Acids Res..

[58] Meer M.V., Podolskiy D.I., Tyshkovskiy A., Gladyshev V.N. (2018). A Whole Lifespan Mouse Multi-Tissue DNA Methylation Clock. Elife.

[59] Kozak C.A., Rowe W.P. (1980). Genetic Mapping of the Ecotropic Virus-Inducing Locus Akv-2 of the AKR Mouse. J. Exp. Med..

[60] Bhadra M., Howell P., Dutta S., Heintz C., Mair W.B. (2020). Alternative Splicing in Aging and Longevity. Hum. Genet..

[61] Wang Y., Liu J., Huang B., Xu Y.-M., Li J., Huang L.-F., Lin J., Zhang J., Min Q.-H., Yang W.-M. (2015). Mechanism of Alternative Splicing and Its Regulation. Biomed. Rep..

[62] Flint M.S., Bovbjerg D.H. (2012). DNA Damage as a Result of Psychological Stress: Implications for Breast Cancer. Breast Cancer Res..

[63] Meyer F., Engel A.M., Krause A.K., Wagner T., Poole L., Dubrovska A., Peitzsch C., Rothkamm K., Petersen C., Borgmann K. (2022). Efficient DNA Repair Mitigates Replication Stress Resulting in Less Immunogenic Cytosolic DNA in Radioresistant Breast Cancer Stem Cells. Front. Immunol..

[64] Schumacher B., Pothof J., Vijg J., Hoeijmakers J.H.J. (2021). The Central Role of DNA Damage in the Ageing Process. Nature.

[65] Razin A., Cedar H. (1991). DNA Methylation and Gene Expression. Microbiol. Rev..

[66] Bacon E.R., Brinton R.D. (2021). Epigenetics of the Developing and Aging Brain: Mechanisms That Regulate Onset and Outcomes of Brain Reorganization. Neurosci. Biobehav. Rev..

[67] Numata S., Ye T., Hyde T.M., Guitart-Navarro X., Tao R., Wininger M., Colantuoni C., Weinberger D.R., Kleinman J.E., Lipska B.K. (2012). DNA Methylation Signatures in Development and Aging of the Human Prefrontal Cortex. Am. J. Hum. Genet..

[68] Cunnane S.C., Trushina E., Morland C., Prigione A., Casadesus G., Andrews Z.B., Beal M.F., Bergersen L.H., Brinton R.D., de la Monte S. (2020). Brain Energy Rescue: An Emerging Therapeutic Concept for Neurodegenerative Disorders of Ageing. Nat. Rev. Drug Discov..

[69] Raichle M.E., Gusnard D.A. (2002). Appraising the Brain’s Energy Budget. PNAS.

[70] Lust W.D., Pundik S., Zechel J., Zhou Y., Buczek M., Selman W.R. (2003). Changing Metabolic and Energy Profiles in Fetal, Neonatal, and Adult Rat Brain. Metab. Brain Dis..

[71] Rogne T., Engstrøm A.A., Jacobsen G.W., Skranes J., Østgård H.F., Martinussen M. (2015). Fetal Growth, Cognitive Function, and Brain Volumes in Childhood and Adolescence. Obstet. Gynecol..

[72] Bonnin A., Goeden N., Chen K., Wilson M.L., King J., Shih J.C., Blakely R.D., Deneris E.S., Levitt P. (2011). A Transient Placental Source of Serotonin for the Fetal Forebrain. Nature.

[73] Bronson S.L., Bale T.L. (2016). The Placenta as a Mediator of Stress Effects on Neurodevelopmental Reprogramming. Neuropsychopharmacology.

[74] Rosenfeld C.S. (2020). Placental Serotonin Signaling, Pregnancy Outcomes, and Regulation of Fetal Brain Development†. Biol. Reprod..

[75] Zeltser L.M., Leibel R.L. (2011). Roles of the Placenta in Fetal Brain Development. Proc. Natl. Acad. Sci. USA.

[76] Barton R.A., Capellini I. (2011). Maternal Investment, Life Histories, and the Costs of Brain Growth in Mammals. Proc. Natl. Acad. Sci. USA.

[77] Lindsay K.L., Buss C., Wadhwa P.D., Entringer S. (2019). The Interplay Between Nutrition and Stress in Pregnancy: Implications for Fetal Programming of Brain Development. Biol. Psychiatry.

[78] Ojeda D.A., Hutton O., Hopkins R., Cagampang F., Smyth N.R., Fleming T.P., Eckert J., Willaime-Morawek S. (2023). Preimplantation or Gestation/Lactation High-Fat Diet Alters Adult Offspring Metabolism and Neurogenesis. Brain Commun..

[79] Singh S.K., Kagalwala M.N., Parker-Thornburg J., Adams H., Majumder S. (2008). REST Maintains Self-Renewal and Pluripotency of Embryonic Stem Cells. Nature.

[80] Zullo J.M., Drake D., Aron L., O’Hern P., Dhamne S.C., Davidsohn N., Mao C.-A., Klein W.H., Rotenberg A., Bennett D.A. (2019). Regulation of Lifespan by Neural Excitation and REST. Nature.

[81] Lind M.I., Spagopoulou F. (2018). Evolutionary Consequences of Epigenetic Inheritance. Heredity.

[82] Hou C. (2013). The Energy Trade-off between Growth and Longevity. Mech. Ageing Dev..

[83] Park S.E., Kim H.S., Kwon S.J., Kim M.-J., Choi S., Oh S., Ryu G.H., Jeon H.B., Na D.L., Chang J.W. (2021). Exposure of Mesenchymal Stem Cells to an Alzheimer’s Disease Environment Enhances Therapeutic Effects. Stem Cells Int..

[84] Srivastava R., Li A., Datta T., Jha N.K., Talukder S., Jha S.K., Chen Z.-S. (2022). Advances in Stromal Cell Therapy for Management of Alzheimer’s Disease. Front. Pharmacol..

[85] Regmi S., Liu D.D., Shen M., Kevadiya B.D., Ganguly A., Primavera R., Chetty S., Yarani R., Thakor A.S. (2022). Mesenchymal Stromal Cells for the Treatment of Alzheimer’s Disease: Strategies and Limitations. Front. Mol. Neurosci..

[86] Crouch J., Shvedova M., Thanapaul R.J.R.S., Botchkarev V., Roh D. (2022). Epigenetic Regulation of Cellular Senescence. Cells.

[87] Baker D.J., Petersen R.C. (2018). Cellular Senescence in Brain Aging and Neurodegenerative Diseases: Evidence and Perspectives. J. Clin. Investig..

[88] Meyers C.A., Albitar M., Estey E. (2005). Cognitive Impairment, Fatigue, and Cytokine Levels in Patients with Acute Myelogenous Leukemia or Myelodysplastic Syndrome. Cancer.

[89] Williams A.M., van Wijngaarden E., Seplaki C.L., Heckler C., Weber M.T., Barr P.M., Zent C.S., Janelsins M.C. (2020). Cognitive Function in Patients with Chronic Lymphocytic Leukemia: A Cross-Sectional Study Examining Effects of Disease and Treatment. Leuk. Lymphoma.

[90] Tylee D.S., Kawaguchi D.M., Glatt S.J. (2013). On the Outside, Looking in: A Review and Evaluation of the Comparability of Blood and Brain “-Omes”. Am. J. Med. Genet. Part B Neuropsychiatr. Genet..

[91] Walton E., Hass J., Liu J., Roffman J.L., Bernardoni F., Roessner V., Kirsch M., Schackert G., Calhoun V., Ehrlich S. (2016). Correspondence of DNA Methylation Between Blood and Brain Tissue and Its Application to Schizophrenia Research. Schizophr. Bull..

[92] Edgar R.D., Jones M.J., Meaney M.J., Turecki G., Kobor M.S. (2017). BECon: A Tool for Interpreting DNA Methylation Findings from Blood in the Context of Brain. Transl. Psychiatry.

[93] Farré P., Jones M.J., Meaney M.J., Emberly E., Turecki G., Kobor M.S. (2015). Concordant and Discordant DNA Methylation Signatures of Aging in Human Blood and Brain. Epigenetics Chromatin.

[94] Lin D., Chen J., Ehrlich S., Bustillo J.R., Perrone-Bizzozero N., Walton E., Clark V.P., Wang Y.-P., Sui J., Du Y. (2018). Cross-Tissue Exploration of Genetic and Epigenetic Effects on Brain Gray Matter in Schizophrenia. Schizophr. Bull..

[95] Strawn M., Safranski T.J., Behura S.K. (2023). Does DNA Methylation in the Fetal Brain Leave an Epigenetic Memory in the Blood?. Gene.

[96] Tollervey J.R., Wang Z., Hortobágyi T., Witten J.T., Zarnack K., Kayikci M., Clark T.A., Schweitzer A.C., Rot G., Curk T. (2011). Analysis of Alternative Splicing Associated with Aging and Neurodegeneration in the Human Brain. Genome Res..

[97] Takata A., Matsumoto N., Kato T. (2017). Genome-Wide Identification of Splicing QTLs in the Human Brain and Their Enrichment among Schizophrenia-Associated Loci. Nat. Commun..

[98] Mazin P., Xiong J., Liu X., Yan Z., Zhang X., Li M., He L., Somel M., Yuan Y., Phoebe Chen Y.-P. (2013). Widespread Splicing Changes in Human Brain Development and Aging. Mol. Syst. Biol..

