# Supplementary material for: Molecular Regulation of Fetal Brain Development in Inbred and Congenic Mouse Strains Differing in Longevity
Source: Genes (Basel). 2024 May 9;15(5):604. doi: 10.3390/genes15050604 (PMC11121069; doi:10.3390/genes15050604)

Supplementary Figure S1. Volcano plots showing significantly upregulated genes (orange) or downregulated genes (green) between developmental times in each strain. The title on the top of each plot shows the time points (d12, d15 or d17) in the strain (A for AKR/J, B for C57BL/6J and C for B6.Cg-*Cav1*^{tm1Mls}/J (*Cav1*-null) mice. The *x*-axis represents fold changes in gene expression in log scale, and *y*-axis represent the significance of differential expression (false discovery rate) in log scale.

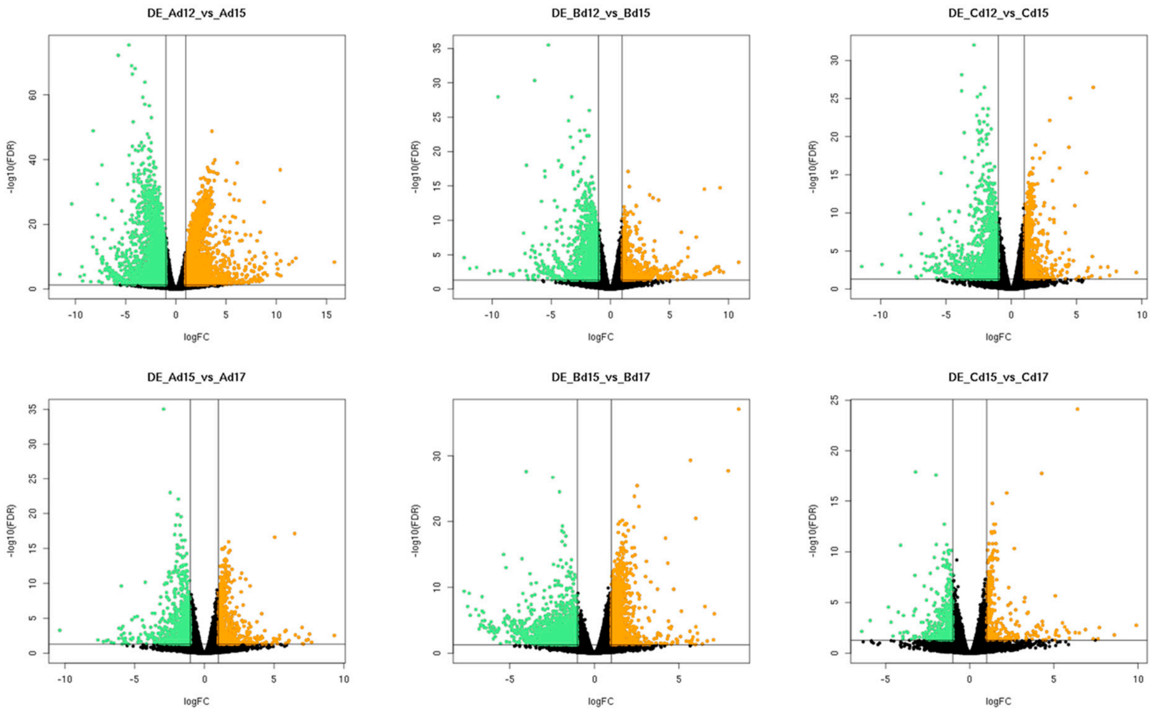

Supplementary Figure S2. Genome browser view of SNPs identified in close proximity (< 100 bp) of CpG sites differentially methylated between the strains. The strains are shown as A for AKR/J, B for C57BL/6J and C for B6.Cg-*Cav1*^{tm1Mls}/J (*Cav1*-null). The methylation positions are shown as chromosome:coordinate below the genes. The SNPs are shown with rs number below the methylation sites. The strains compared are shown on the left of the plots.

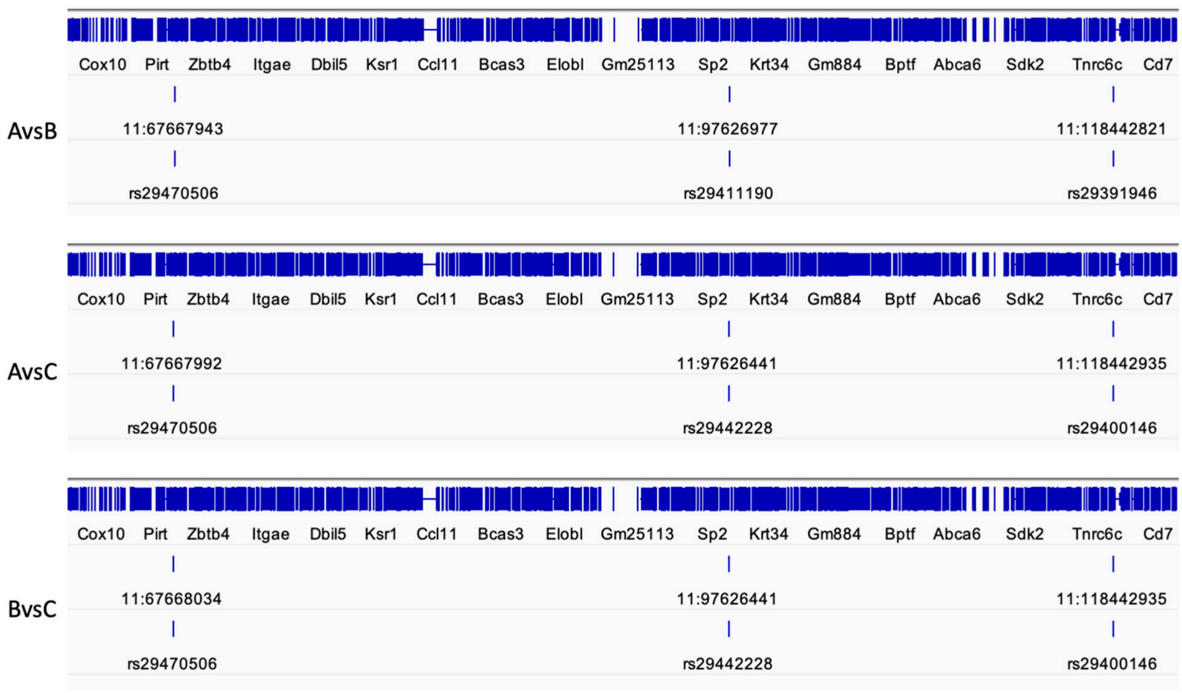

Supplement: Supplementary file 1 [file genes-15-00604-s001.zip › Supplementary_Figures_with_legends.pdf]
